# CycP: A Novel Self-Assembled Vesicle-Forming Cyclic Antimicrobial Peptide to Control Drug-Resistant *S. aureus*

**DOI:** 10.3390/bioengineering11080855

**Published:** 2024-08-21

**Authors:** Piyush Baindara, Dinata Roy, Santi M. Mandal

**Affiliations:** 1 Animal Sciences Research Center, Division of Animal Sciences, University of Missouri, Columbia, MO 65211, USA; pbaindara@missouri.edu; 2 Department of Zoology, Mizoram University, Aizawl 796004, India; dinataroy9@gmail.com; 3 Department of Bioscience and Biotechnology, Indian Institute of Technology Kharagpur, Kharagpur 721302, India; 4 Department of Chemistry and Biochemistry, University of California San Diego, 9500 Gilman Dr, La Jolla, CA 92093, USA

**Keywords:** antimicrobial peptides, peptide vesicles, drug delivery, antibiotic resistance

## Abstract

Antimicrobial peptides (AMPs) are considered a promising alternative to conventional antibiotics to fight against the rapid evolution of antibiotic resistance. Other than their potent antimicrobial properties, AMP-based vesicles can be used as efficient drug-delivery vehicles. In the present study, we synthesized and characterized a new cyclic AMP, consisting of all-hydrophobic cores with antimicrobial activity against *S. aureus*. Interestingly, CycP undergoes supramolecular self-assembly, and self-assembled CycP (sCycP) vesicles are characterized under an electron microscope; however, these vesicles do not display antimicrobial activity. Next, sCycP vesicles are used in combination with SXT (sulfamethoxazole–trimethoprim) vesicles to check the drug loading and delivery capacity of sCycP vesicles to bacterial cell membranes. Interestingly, sCycP vesicles showed synergistic action with SXT vesicles and resulted in a significant reduction in MIC against *S. aureus*. Further, electron microscopy confirmed the membrane-specific killing mechanism of SXT-loaded sCycP vesicles. Additionally, CycP showed high binding affinities with the β-lactamase of *S. aureus*, which was one of its possible antimicrobial mechanisms of action. Overall, the results suggested that CycP is a novel self-assembled dual-action cyclic AMP with non-cytotoxic properties that can be used alone as an AMP or a self-assembled drug delivery vehicle for antibiotics to combat *S. aureus* infections.

## 1. Introduction

AMPs are well known for their favorable properties to be considered as alternatives to conventional antibiotics in the fight against antibiotic-resistant pathogens [1]. However, large size, rapid degradation, and delivery issues are some major drawbacks in the way AMPs are used in clinical settings. By considering the AMP roadmap in the recent past, serious efforts are required to overcome the issues associated with AMPs to be used in clinical settings. In the same pathway, self-assembled AMPs are a new hope in the field of AMP-based drug delivery and nanomedicine [2]. It has been reported and known that small peptides can adopt various assemblies such as vesicles, droplets, nanotubes, and bi-lamellar sheets [3,4,5]. AMPs have also been seen to self-assemble into nanospherical forms or vesicles with hollow and solid cores [6]. AMP-based vesicles and scaffolds are highly significant due to their easy synthesis and high yields, well-defined structure, biocompatibility, diversity, and properties that can be further tuned by bioengineering [7,8,9,10]. In a recent study, branched amphiphilic peptides were demonstrated to form self-assembled nanovesicles that can trap fluorescent dye molecules within their interior, and were suggested as efficient drug delivery candidates [7]. Next, peptide-based polyion complex vesicles are shown to deliver neomycin phosphotransferase II (NPTII), a kanamycin resistance enzyme, to the root hair cells of *Arabidopsis thaliana* to fight antibiotic resistance without genetic modification [11]. In another study, peptide-based self-assembled nanostructures used in photodynamic cancer therapy suggested the diverse potential of self-assembled peptide structures [12]. In addition to this, AMP-based vesicles can be used as dual-purpose drug delivery systems where AMPs have direct antimicrobial activity along with the loading and delivery of other antimicrobial substances. In the present study, we have synthesized a novel all-hydrophobic core cyclic antimicrobial peptide. This newly synthesized cyclic peptide not only displayed direct antimicrobial activity against drug-resistant *S. aureus* but also formed self-assembled vesicles that carry antibiotics within it, resulting in enhanced activity.

To the best of our knowledge, this is the first time we are reporting a novel cyclized AMP with a vesicle-based delivery of antibiotics in a somewhat uncommon way. This AMP-based self-assembled vesicle drug delivery method could be further developed as a potential drug delivery system in clinical or therapeutic settings.

## 2. Materials and Methods

### 2.1. Solid-Phase Peptide Synthesis, Purification, and Molecular Weight Determination

All linear peptidyl-resin precursors for the cyclic peptide were synthesized employing Fmoc-SPPS (Solid Phase Peptide Synthesis) on TentaGel XV RAM resin (Sigma-Aldrich, Burlington, MA, USA) using a Biotage Syro I automated parallel peptide synthesizer. Fmoc-protected amino acids were employed to perform coupling reactions using HBTU or HOBt in the presence of 0.4 M N-methylmorpholine (NMM) in Dimethylformamide (DMF). Next, Fmoc-deprotection cycles are carried out using 20% piperidine in DMF. Cysteine side-chain-protecting groups were removed in situ during the final cyclization step to form the end-to-end disulfide bridge in the presence of I_2_, DMSO, and anisole in DMF [13]. The synthesized peptide was purified by RP-HPLC (Agilent Infinity 1260, Santa Clara, CA, USA) equipped with a fraction collector, using a C18 column (Zorbax 300SB-C18 7 µm 21.2 × 250 mm) at 220 nm. Mobile phases consisting of water with 0.12% trifluoroacetic acid (TFA; mobile phase A) and acetonitrile with 0.1% TFA (ACN; mobile phase B) were used over 30 min at a flow rate of 15 mL/min [14]. The molecular weight of the purified peptide was determined using an Agilent LC system equipped with an Agilent 6510 Q-TOF mass spectrometer (Agilent, Santa Clara, CA, USA). All the chemicals and HPLC-grade solvents were purchased from Fisher Scientific, MA, USA, or Sigma-Aldrich, MA, USA.

### 2.2. Multiple Sequence Alignment and De Novo Peptide Structure Prediction

The synthesized 13-amino-acid-long sequence of cyclic peptide CycP was used to perform a similarity search on the APD3 antimicrobial peptide database (https://aps.unmc.edu/AP) (accessed on 6 February 2024) [15]. The similarity percentage of CycP with known AMPs was analyzed by multiple sequence alignment, using BioEdit version 7.2.5. Next, to check the helical or non-helical probabilities of peptides, a 2D helical wheel was generated using the helical wheel tool at Galaxy CPT Public (https://cpt.tamu.edu/galaxy-pub) (accessed on 6 February 2024). Further, to acquire the 3D structure of CycP, de novo peptide structure was predicted using PEP-FOLD 2.0 at the RPBS web portal (https://mobyle.rpbs.univ-paris-diderot.fr/cgi-bin/portal.py#forms::PEP-FOLD) (accessed on 6 February 2024). The 3D structure of CycP was further refined using the Galaxy Refine server (http://galaxy.seoklab.org/cgi-bin/submit.cgi?type=REFINE) (accessed on 6 February 2024) [16]. The final validation of the CycP 3D structure was performed using the SAVES v6.0 server (https://saves.mbi.ucla.edu/results?job=1654027&p=procheck) (accessed on 6 February 2024) that defined the quality of the modeled tertiary structure of CycP by the percentage of residues in allowed and disallowed regions.

### 2.3. Bacterial Strain and Media

To check and compare the antimicrobial potential of the newly synthesized peptide, a clinically isolated drug-resistant strain of *S. aureus* was obtained from the Department of Microbiology, Bankura Sammilani Medical College and Hospital, Kenduadihi, Bankura 722102, West Bengal, India. The strain was primarily isolated and identified as methicillin-resistant *S. aureus*. The target test strain of *S. aureus* was grown on nutrient agar (NA) and nutrient broth (NB) for single colonies and liquid cultures, respectively. All bacterial mediums were purchased from Hi Media, Mumbai, Maharashtra, India.

### 2.4. Peptide Self-Assembly and Vesicle Preparation

To prepare the peptide vesicles, CycP was dissolved in an equal ratio of acetonitrile and methanol at 1 mg/mL concentration in a glass vessel. Subsequently, the peptide solution was rotated in chloroform for 4–5 h at 50 °C, allowing the self-assembly of peptides in a non-polar environment created by CHCl_3_ [17]. On the other hand, sulfamethoxazole–trimethoprim (SXT) was dissolved in 70% ethanol under vigorous stirring in a closed glass vessel and subsequently rotated for 1 h in a water bath at 30 °C. This allows SXT vesicle formation in a polar environment created by ethanol. Further, non-polar peptide vesicles were mixed with polar vesicles of SXT and spun for 4 h at room temperature for the formation of SXT-loaded peptide vesicles. Finally, the mixture was dialyzed in water using a 7 kDa membrane cassette (Fisher Scientific, Waltham, MA, USA) to remove any traces of solvent. Acetonitrile, chloroform, ethanol, and methanol were purchased from Fisher Scientific, USA.

### 2.5. Minimum Inhibitory Concentration Determination

The minimum inhibitory concentrations (MICs) for CycP, sCycP vesicles, SXT, and the combination of sCycP with SXT were determined using a 96-well microtiter plate dilution assay [18]. Different dilutions of CycP, sCycP vesicles, and SXT and a combination of sCycP with SXT were prepared using the synthesized and HPLC-purified dry powder in PBS. Target strain *S. aureus* (~2 × 10^4^ cells) was treated with different concentrations of the CycP, sCycP vesicles, and SXT at various concentrations for 24 h, while OD was observed every 2 h of the time interval. PBS alone and SXT were used as controls. All the tests were performed three times independently in triplicate to analyze the final results. The antibiotic mixture, SXT, and PBS were purchased from Hi Media, India.

### 2.6. SEM Sample Preparation for Peptide Vesicles

For SEM sample preparation, 10 µL of CycP vesicle solution was directly placed on a copper grid and allowed to dry for 10 min. Next, the peptide sample was fixed in a modified Karnovsky fixative for 2 h. As the peptide vesicles were non-conducive, samples were further fixed with a drop of 2% OsO_4_ (Sigma Aldrich, USA) for 10 min in a covered Petri dish and then gradually dehydrated in graded ethanol (30–100%). Finally, ethanol-dehydrated samples were freeze-dried overnight before mounting on aluminum stubs for imaging [18]. The mounted CycP samples were photographed under a ZEISS EVO 60 scanning electron microscope with an Oxford EDS detector (Zeiss, Dublin, CA, USA).

### 2.7. TEM Sample Preparation for Bacterial Cells

A mid-exponential stage culture of *S. aureus* was centrifuged at 8000× *g* for 10 min, and cells were collected and washed thrice with PBS (Hi Media, India). The washed cell pellet was diluted with PBS to a cell density of approximately 10^8^ CFU mL^−F^ and subsequently treated with a suboptimal concentration (32 µg/mL) of the combination mixture of sCycP and SXT vesicles for 30 min at 37 °C. After treatment, bacterial cells were washed with PBS and then fixed in modified Karnovsky fixative. Then, 1% osmium tetroxide (Sigma-Aldrich, USA) in 100 mM PBS was further used to fix the bacterial cells and then subsequently embedded in 2% agarose. ACM I and ACM II (Sigma-Aldrich, USA) were used to make blocks, and sectioning was performed using an ultramicrotome (Leica EM UC7, IL, USA) [14]. PBS-treated bacterial cells were used as a control. Finally, sections (70 nm) containing bacterial cells were stained with 0.1% (*w*/*v*) PTA (Phosphotungstic acid, Sigma-Aldrich, Burlington, MA, USA) on a carbon-coated copper grid (300 mess, Polysciences, Warrington, PA, USA) and analyzed under TEM at a resolution of 0.2–0.5 µM (JEM 2100F, JEOL, Tokyo, Japan).

### 2.8. Molecular Docking

The crystal structure of β-lactamase from *S. aureus* PC1 (PDB ID: 3BLM) was retrieved in PDB format from the RCSB PDB database (http://www.rcsb.org) (accessed on 6 February 2024) to explore CycP interaction with it. Further, the structure modification of β-lactamase was carried out by removing preoccupied ligands, ions, and water molecules by using Chimera 1.15 (https://www.cgl.ucsf.edu/chimera/) (accessed on 6 February 2024), and active site residues were predicted by using an online tool, Fpocket (https://bioserv.rpbs.univ-paris-diderot.fr/services/fpocket/) (accessed on 6 February 2024) [19].

Molecular docking experiments were performed by using the HDOCK online server http://hdock.phys.hust.edu.cn/ (accessed on 6 February 2024) [20]. The best protein–peptide complex was selected from among the top ten conformers based on docking scoring, while interaction analysis was conducted using PyMOL and Discovery Studio [21,22].

### 2.9. Cell Toxicity Assay

The cell toxicity of CycP and sCycP vesicles was analyzed by an MTT assay using the human cervical cancer cell line (HeLa), human breast cancer cell line (MCF-7), and human lung carcinoma cell line (H1299). All the cells were grown in DMEM medium (Invitrogen, USA) supplemented with 10% fetal bovine serum (Invitrogen, Waltham, MA, USA) and a 1% penicillin–streptomycin cocktail (Sigma, Livonia, MI, USA) while maintained in a CO_2_ incubator (Thermo Scientific, USA) at 37 °C and 5% CO_2_ environment. All the cell lines were purchased from the American Type Culture Collection (ATCC, Manassas, VA, USA). For the assay, all three cell lines were seeded (5 × 10^3^ cells/well) in 96-well plates. The growth medium was replaced after 24 h of incubation with fresh medium containing different concentrations of CycP (0–256 μg/mL), and sCycP vesicles (0–1 mg/mL). The growth medium was used as a negative control, while 1% Triton X-100 (G Biosciences, St. Louis, MO, USA) served as a positive control. The treated cells were incubated for 24 h and then 20 μL of MTT solution (5 mg/mL in PBS) was added to each well. Cells were further incubated with MTT solution for 3 h at 37 °C and 50 μL of dimethyl sulfoxide (Sigma, USA) was added to each well after the removal of the MTT-containing medium. Finally, absorbance was recorded at 590 nm on an ELISA plate reader (Thermo Scientific, USA). Absorbance was represented as % viable cells, while untreated cells were considered as 100% viable.

### 2.10. Statistical Analysis

In this study, each respective outcome is based on the mean ± standard deviation of the mean (SD). Parametric data analysis involves the use of two-way analysis of variance (ANOVA) and the Bonferroni post-test procedure for group comparison. The D’Agostino & Pearson omnibus normality test and the one-sample *t*-test were used to examine column statistics for non-parametric data. *p* < 0.05 in each experiment is the threshold for significance for the final results of each experiment. Each non-parametric data experiment was run in triplicate, three times separately.

## 3. Results

### 3.1. CycP Is a Novel Cyclic Peptide

**Figure 1:**
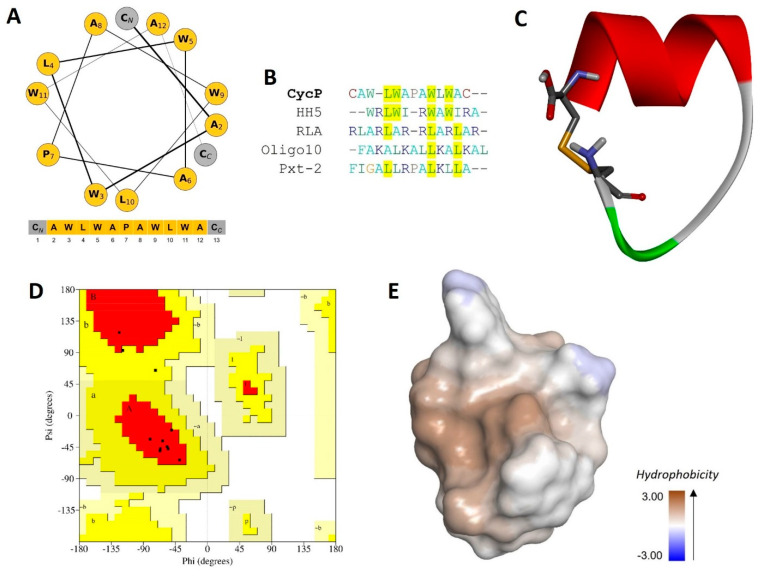
Synthesis, characterization, and antimicrobial activity of a novel cyclic AMP, CycP. (**A**) Helical wheel representation of CycP (13 amino acids). All hydrophobic amino acids are shown in yellow color, while cysteine residues are shown in gray color at both N and C terminals. (**B**) Multiple sequence alignment of CycP with other known AMPs. Common amino acids are highlighted in yellow. (**C**) The 3D structure of CycP is end-to-end cyclized via a disulfide bond (represented as sticks). (**D**) Ramachandran plot for CycP 3D structure validation. Most favored regions are shown in red color, while additional allowed regions are shown in yellow color. (**E**) The 3D surface structure of CycP is completely hydrophobic.

The solid-phase synthesis of a novel cyclic peptide, CycP, is carried out in the present study. We designed the peptide sequence with all-hydrophobic core amino acids with a polar cysteine residue at both the N and C terminus of the peptide (Figure 1A). The final output of peptide synthesis is a 13-amino-acid-long peptide that is cyclized by a disulfide bond. The synthesized peptide is further purified and characterized using RP-HPLC to determine the molecular weight and confirmation of the disulfide bond (Figures S1 and S2). A single peak collected from HPLC fractions showed a molecular mass of 1574.10 (M-H), which is 2Da less than the calculated molecular weight (1576.90 Da) of the peptide sequence using the Expasy ProtParam tool (https://web.expasy.org/protparam) (accessed on 6 February 2024) (Figure S2). We performed a sequence similarity search using the APD3 database to check the novelty of the newly synthesized peptide (Figure 1B). The sequence alignment revealed only 40% similarity to the HH5 synthetic peptide (APD ID: AP03954), which confirms the novelty of CycP. To have a look into the secondary structure of CycP, we made a helical wheel using the amino acid sequence that confirmed the presence of a helix in the peptide structure. Further, to obtain more details about the CycP 3D structure, we predicted the de novo 3D structure of the peptide employing the PEP-FOLD 2.0 server (Figure 1C). A Ramachandran plot analysis of the predicted structure revealed 80.0% residues in the most favored regions and 20.0% residues in additional allowed regions (Figure 1D). The results showed a high-quality cyclic helical structure for the CycP with an all-hydrophobic surface (Figure 1C,E).

### 3.2. CycP Forms Self-Assembled Peptide Vesicles

CycP is designed as a cyclic peptide with all-hydrophobic core amino acid sequences. We checked the self-assembled vesicle formation properties of CycP in the presence of chloroform that provides a non-polar environment [23]. CycP is used at a concentration of 1 mg/mL for the vesicle formation experiment. Following the vesicle formation procedure, we performed the SEM experiment to check the presence of vesicles. SEM images confirmed the self-assembled vesicle formation by CycP with an average diameter of 193.5 ± 20 nm measured during the live capture of SEM images (Figure S3).

### 3.3. CycP Is a Dual-Action Cyclic AMP

**Figure 2:**
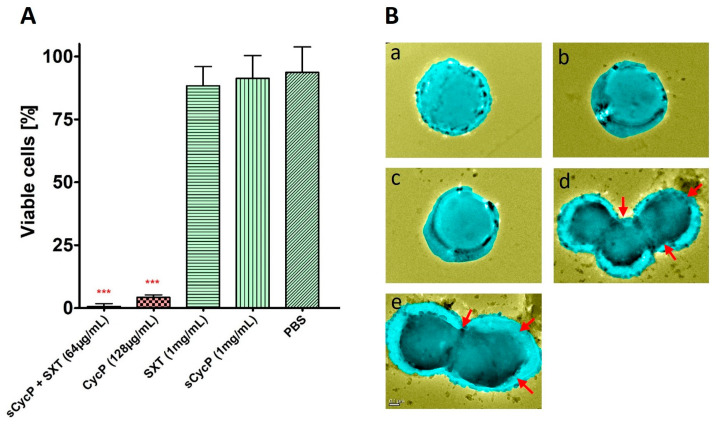
Antimicrobial potential of CycP and sCycP vesicles with SXT. (**A**) MICs for CycP and sCycP vesicles with SXT against *S. aureus*. PBS is used as a control. Error bars show a standard deviation (SD), while the statistical significance is considered at the level of *p* < 0.05 (indicated as red stars above bars). The experiment was performed three times independently in triplicate. (**B**) TEM images of *S. aureus* treated with PBS (**a**), sCycP vesicles (**b**), SXT (**c**), CycP (**d**), and sCycP vesicles loaded with SXT (**e**). Red arrows indicate the cell clumping, shrinkage, and lysis. Images are artificially colorized for better visualization of cell damage upon drug treatment.

A 96-well microtiter plate dilution assay is performed to check the antimicrobial potential of CycP against a drug-resistant strain of *S. aureus*, which is confirmed to have resistance against SXT at a high concentration of 1 mg/mL (Figure 2A). Different dilutions of CycP in PBS ranging from 8 to 128 µg/mL are used to determine the MIC values. The results confirmed that CycP was able to kill the drug-resistant *S. aureus* at a concentration of 128 µg/mL, which was too high (Figure 2A). As CycP can form self-assembled vesicles, we performed the MIC assay for sCycP vesicles; however, the results displayed no activity, even at 1 mg/mL of vesicle concentration (Figure 2A). Further, to check the antibiotic loading and carrying capacity of sCycP vesicles, we prepared the SXT vesicles in a polar environment (ethanol) and mixed them with the non-polar sCycP vesicles as described in the methods. The loading of SXT vesicles is assumed within the sCycP vesicles due to the interaction and fusion between non-polar and polar vesicles. To determine the MIC values of antibiotic-loaded sCycP vesicles, different dilutions in PBS were used in the range of 8–128 µg/mL against *S. aureus*. Interestingly, antibiotic-loaded sCycP vesicles revealed a MIC of 64 µg/mL, which was 50% less than the MIC values of CycP alone (Figure 2A).

**Figure 3:**
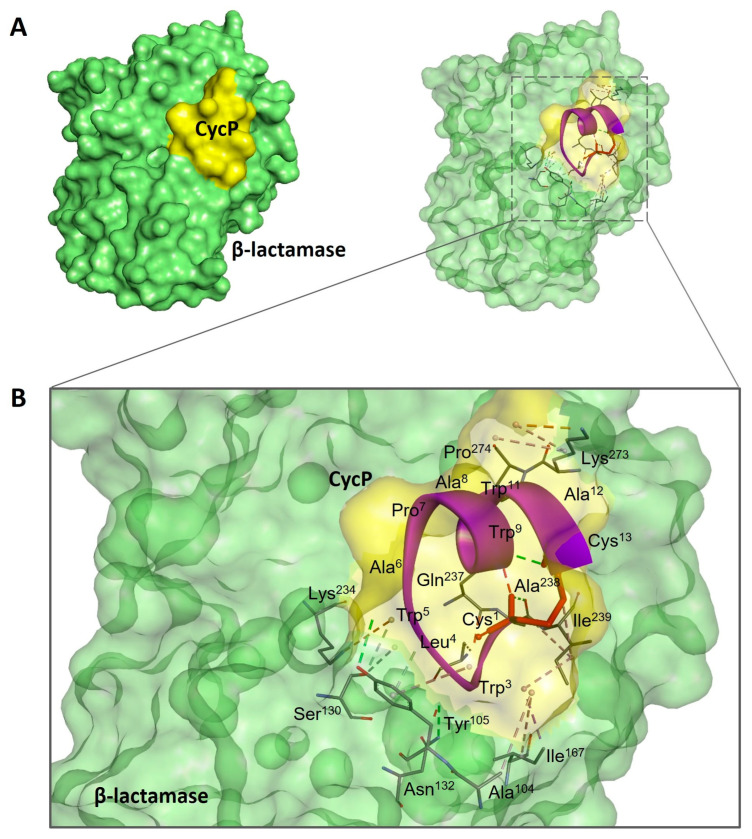
Molecular docking analysis of CycP with β-lactamase of *S. aureus*. (**A**) The docked complex of CycP with β-lactamase represents a solid surface model where CycP is shown in yellow color and β-lactamase is shown in green color. (**B**) A zoomed-in image of the docked complex of CycP and β-lactamase with interacting amino acid residues. CycP is shown as a solid purple ribbon overlapping with the transparent docked surface of CycP. The disulfide bond between Cys1 and Cys13 of CycP is shown as a red stick. Interactive amino acid residues at the binding site are shown as solid sticks in various colors, while the bonds are represented as dotted lines in different colors.

To further explore the antimicrobial potential of CycP against *S. aureus*, we performed a molecular docking experiment of CycP with β-lactamase of *S. aureus*. Interestingly, CycP showed an efficient binding affinity with β-lactamase, revealing a high binding free energy of −211.06 kcal (Figure 3A,B). In conclusion, CycP is a self-assembled cyclic dual-action AMP that can kill the drug-resistant *S. aureus* directly by membrane-specific killing, including blocking β-lactamase, and can also carry the antibiotic within the self-assembled vesicles to deliver the target bacterial cell membrane for subsequent killing.

### 3.4. Self-Assembled CycP Vesicles Deliver the Antibiotic and Kill the Drug-Resistant S. aureus in a Membrane-Specific Mechanism of Action

Drug-resistant *S. aureus* is completely eradicated within 24 h of SXT-loaded sCycP vesicle treatment at a dosage of 64 µg/mL, as determined in the MIC assay. To observe any interactions with the *S. aureus* cell membrane or morphological changes, we conducted the TEM experiment with the suboptimal concentration of SXT-loaded sCycP vesicles at 32 µg/mL for a 30 min treatment time. TEM images showed PBS-treated control *S. aureus* cells as distinct cocci with smooth, undamaged surfaces. Conversely, *S. aureus* treated with SXT-loaded sCycP vesicles at a 32 µg/mL dose revealed an aberrant cell shape, including disrupted cell membranes that resulted in shrunken, flattened, and lysed cell aggregates (Figure 2B). These results were in agreement with the MIC experiment against sCycP vesicles against *S. aureus*. Overall, the TEM data demonstrated a cell membrane-specific killing mechanism of action by SXT-loaded sCycP vesicles that demonstrated as damaged, lysed, and clumped cells which ultimately leads to death.

### 3.5. CycP and sCycP Vesicles are Non-Cytotoxic

**Figure 4:**
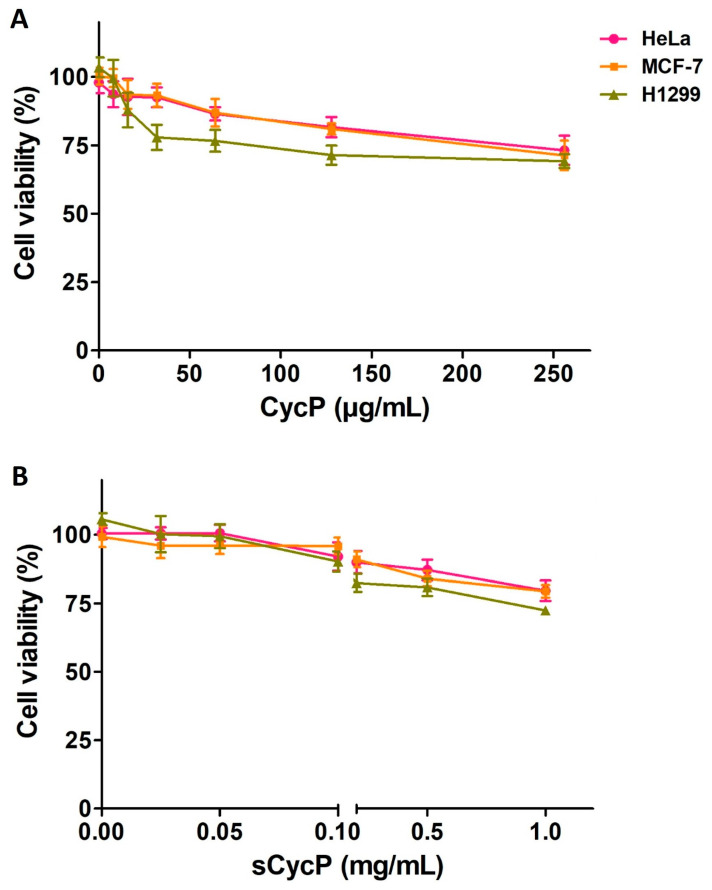
CycP and sCycP vesicles are non-cytotoxic. (**A**) MTT assay for CycP (**A**), and (**B**) sCycP vesicles against HeLa, MCF-7, and H1299 cell lines. PBS and 1% Triton X-100 were used as negative and positive controls, respectively. Error bars show the standard deviation (SD), while the statistical significance is considered at the level of *p* < 0.05. The experiment was performed three times independently in triplicate.

Both CycP and sCycP vesicles loaded with SXT were found to have efficient activity against drug-resistant *S. aureus*. However, having non-cytotoxic properties is essential for an antimicrobial agent to be used in clinical settings. First, to analyze the non-cytotoxic nature of CycP, we employed the ToxinPred server [24]. The screening of the ToxinPred server suggested CycP as a non-cytotoxic peptide. Next, we performed the cytotoxicity assay using CycP and sCycP vesicles against human cell lines, HeLa, MCF-7, and H1299. Interestingly, CycP and sCycP vesicles were both found not cytotoxic against all three cell lines. More than 70% of cells were found viable even at a 256 µg/mL concentration of CycP and 1 mg/mL concentration of sCycP vesicles after 24 h of treatment (Figure 4A,B). Overall, the in silico and in vitro results suggested that both CycP and sCycP vesicles are non-cytotoxic against human cell lines and could be used for therapeutic applications in clinical settings.

## 4. Discussion

AMPs are suggested as one of the potential alternatives to traditional antibiotics in the fight against the rapid emergence of drug-resistant bacteria. Although AMPs possess interesting properties to be a much sought-after candidate for therapeutic development, their size and thus efficient delivery in clinical settings have always remained a challenge [25]. Using peptide self-assembled vesicles as carriers and for the efficient delivery of antibiotics to a target is a new and interesting approach to using AMPs as dual-action therapeutic candidates. In this way, AMP self-assembled vesicles are not only used for efficient delivery to kill the target bacterial strain but also perform a synergistic antimicrobial action with the cargo that results in enhanced antimicrobial activity against pathogens [26]. Here, in the present study, we synthesized, a 13-amino-acid-long peptide, CycP, with an all-hydrophobic core and end-to-end cyclized via a disulfide bond in the presence of cysteine residues at both N and C terminals (Figure 1A,C). CycP is active against drug-resistant *S. aureus* at a high concentration of 128 µg/mL (Figure 2A). Interestingly, CycP formed self-assembled vesicles in the non-polar environment (Figure S3). However, sCycP vesicles did not display activity against *S. aureus*, even at a high concentration of 1 mg/mL, but could carry and deliver the SXT vesicles. As a result, dual-action sCycP successfully delivered the SXT to drug-resistant *S. aureus* and revealed synergistic killing at 64 µg/mL (Figure 2A). Synergistic killing by SXT-loaded sCycP vesicles is characterized as a membrane-specific mechanism of action using TEM (Figure 2B). Importantly, CycP alone was found effective against *S. aureus* at a concentration of 128 µg/mL, which was also confirmed with TEM (Figure 2B). Interestingly, CycP molecular docking revealed strong affinities of CycP with *S. aureus* β-lactamase, which suggests another possible antimicrobial mechanism of action for CycP (Figure 3A,B). Further, in silico screening along with in vitro experiments demonstrated the non-cytotoxic properties of CycP and sCycP vesicles that further confirmed the therapeutic potential of CycP. Overall, the results demonstrated a dual-action, novel self-assembled vesicle-forming cyclic AMP that can carry and deliver antibiotics to the bacterial cell membrane.

## 5. Conclusions

In summary, the present study reported the synthesis of a novel cyclic AMP with self-assembled vesicle formation capabilities. Also, the peptide vesicles can carry the antibiotic cargo and successfully deliver it to the target, which results in synergistic and efficient killing. In this process, both polar and non-polar solvents are used; in the polar solvent, hydrophobic amino acid residues tend to aggregate, which is energetically unfavorable for them due to their low solubility. This aggregation is driven by hydrophobic interactions, where the hydrophobic residues cluster together to form a core, shielding themselves, and the presence of proline in the peptide sequence introduces a kink or bend in the peptide backbone, leading to the formation of loops, which affects the overall conformation of the cyclic peptide chain to form a vesicle-like structure. Overall, CycP is a novel dual-action self-assembled cyclic AMP that can carry and deliver an antibiotic to the bacterial cell membrane and can also kill drug-resistant *S. aureus*, possibly via a cell membrane-specific killing mechanism and the blocking of β-lactamase.

## 6. Limitations of the Present Study

CycP demonstrated a self-assembled vesicle-forming ability, the delivery of antibiotics to the bacterial cell membrane, and subsequently killing without any cytotoxic effects. However, there are some limitations associated with the present study. More strategies should be devised for more controlled vesicle formation concerning size. The results represented a novel strategy to carry antibiotics in the self-assembled vesicles of AMP; however, further physical and analytical experiments are required to fine-tune this for future applications. Also, CycP is tested only against *S. aureus*, and only the SXT antibiotic mixture is used; however, the use of other test targets and antibiotics may provide more insights about the efficacy and CycP mechanism of action. Furthermore, the in vivo efficacy of CycP and its administration strategies are not examined; therefore, the outcomes may differ in different clinical or experimental settings. Additionally, the interaction of CycP with β-lactamase was interesting; however, further in vitro experiments are required to validate this. Nevertheless, despite these facts and limitations, the current study’s findings offer new hope for the development of AMP-based vesicles and dual-mechanism delivery vehicles to combat the swift evolution of drug-resistant bacteria and new infections, as well as provide hints for future laboratory studies about CycP.

## Data Availability

The original contributions presented in the study are included in the article/supplementary material, further inquiries can be directed to the corresponding author.

## Supplementary Materials

The following supporting information can be downloaded at: https://www.mdpi.com/article/10.3390/bioengineering11080855/s1, Figure S1: RP-HPLC spectra of CycP purification after synthesis. Figure S2: Molecular weight determination of CycP using HPLC purified fractions. Figure S3: Self-assembled vesicles formed by CycP (visualized in SEM).
