# Supplementary material for: CycP: A Novel Self-Assembled Vesicle-Forming Cyclic Antimicrobial Peptide to Control Drug-Resistant S. aureus
Source: Bioengineering (Basel). 2024 Aug 21;11(8):855. doi: 10.3390/bioengineering11080855 (PMC11351190; doi:10.3390/bioengineering11080855)

CycP: A novel self-assembled vesicle-forming cyclic antimicrobial peptide to control drug-resistant *S. aureus*

Piyush Baindara [1], Dinata Roy [2], Santi M. Mandal [3, 4#]

[1]Animal Sciences Research Center, Division of Animal Sciences, University of Missouri, Columbia 65211, MO, USA

[2] Department of Zoology, Mizoram University, Aizawl, Mizoram 796004, India

[3]Department of Bioscience and Biotechnology, Indian Institute of Technology Kharagpur, Kharagpur 721302, WB, India

[4] Department of Chemistry and Biochemistry, University of California San Diego, 9500 Gilman Dr, La Jolla, CA 92093, USA

[#]Correspondence:
Santi M. Mandal, PhD
Department of Chemistry and Biochemistry,
University of California San Diego,
9500 Gilman Dr, La Jolla, CA 92093, USA
Email: mandalsm@gmail.com

Figure S1. RP-HPLC spectra of CycP purification after synthesis.

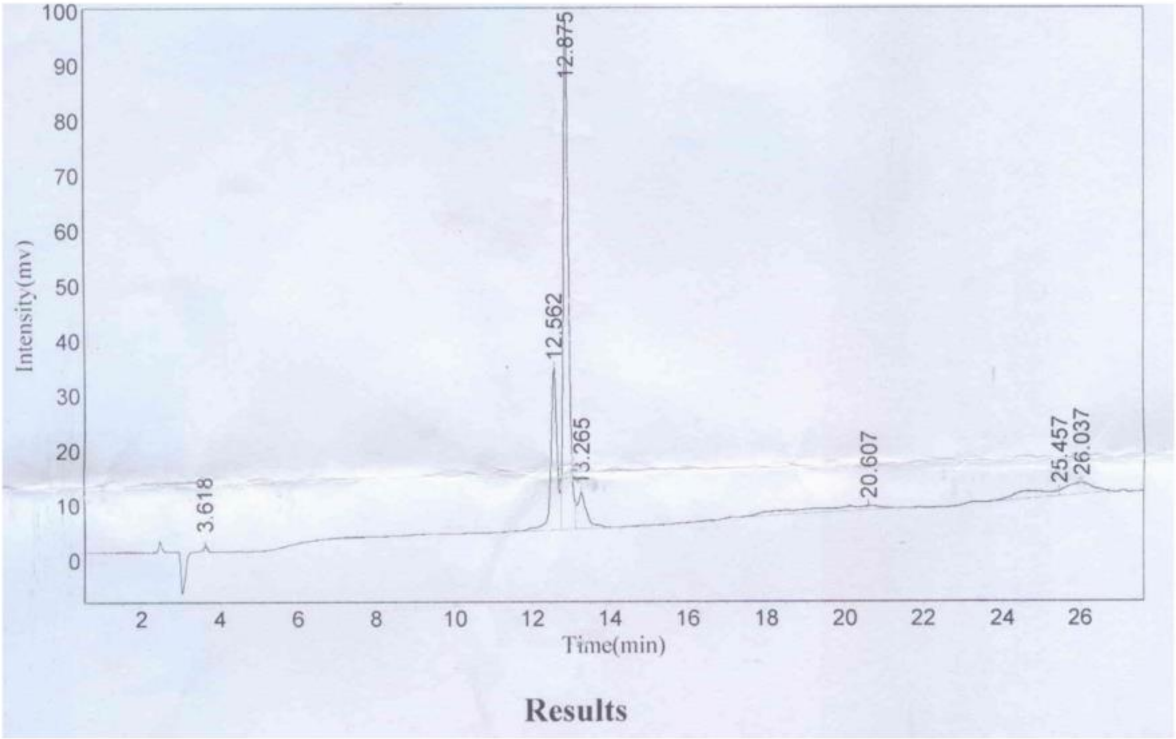

Figure S2. Molecular weight determination of CycP using HPLC purified fractions.

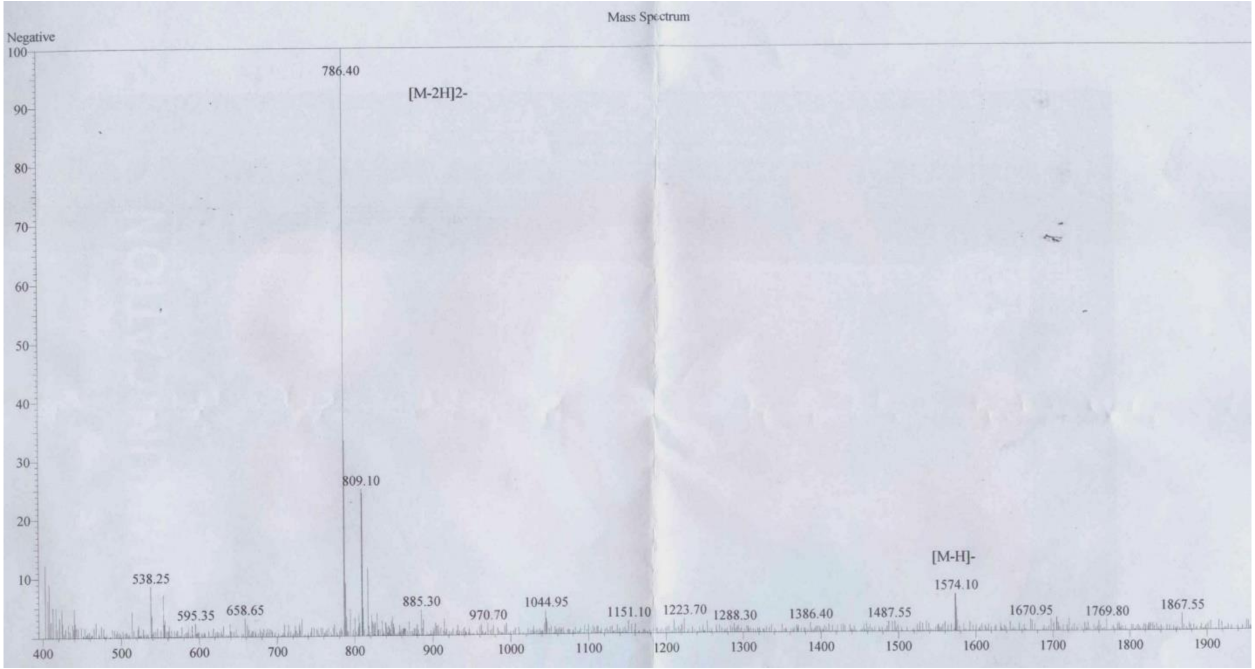

Figure S3. Self-assembled vesicles formed by CycP (visualized in SEM).

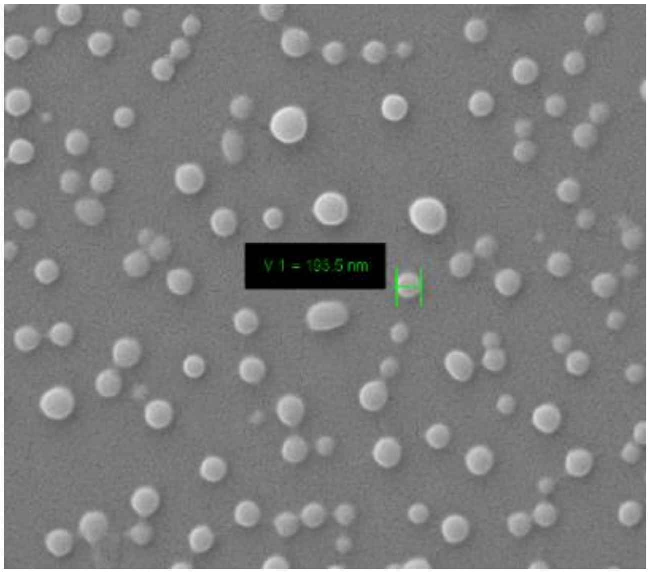

Supplement: Supplementary file 1 [file bioengineering-11-00855-s001.zip › bioengineering-3140526-supplementary.pdf]
